# Neighborhood Environment Perceptions among Latinos in the U.S.

**DOI:** 10.3390/ijerph16173062

**Published:** 2019-08-23

**Authors:** Lilian G. Perez, John M. Ruiz, David Berrigan

**Affiliations:** 1 Cancer Prevention Fellowship Program, National Cancer Institute, Rockville, MD 20850, USA; 2 Department of Psychology, University of Arizona, Tucson, AZ 85721, USA; 3 Division of Cancer Control and Population Sciences, National Cancer Institute, Rockville, MD 20850, USA

**Keywords:** built environment, neighborhood perceptions, acculturation, inequities, Latinos, Hispanics

## Abstract

In the U.S., immigrants and racial/ethnic minorities (e.g., Latinos) often report unfavorable neighborhood environments, which may hinder physical activity (PA). Among Latinos, PA levels are disproportionately lower in foreign-born, female, older, and low-education individuals. It is unclear whether these subgroups, including those from multiple disadvantaged backgrounds (e.g., low education, foreign-born), perceive worse neighborhood environments for PA. This cross-sectional study aimed to examine differences in neighborhood environment perceptions among Latinos in the 2015 National Health Interview Survey (*N* = 4643; 59% foreign-born). Logistic regression models examined nativity—and its interactions with age, gender, and education—in relation to the perceived presence of transportation infrastructure (two items) and destinations (four items), controlling for self-reported walking. Models used sample weights and accounted for the complex survey design. Nativity was not significantly associated with neighborhood environment perceptions. However, nativity interactions with age and education showed the greatest inequities (lowest perceptions) of neighborhood infrastructure (e.g., fewer sidewalks) or destinations (e.g., fewer places to relax) among disadvantaged U.S.-born (older or low education) and advantaged foreign-born (higher education) Latinos. Findings suggest neighborhood perceptions are shaped by complex interactions of nativity with structural (education) and contextual (age) factors. Additional research is needed to complement our findings and inform environmental interventions targeting Latinos.

## 1. Introduction

Residents who report favorable perceptions of their neighborhood environments, such as better infrastructure for walking and having destinations within walking distance, often report higher levels of physical activity (PA) for leisure and transportation [1] and have better health outcomes, such as lower obesity [2]. In contrast, unfavorable neighborhood perceptions may contribute to lower PA and health disparities [2]. In the U.S., Latinos are less likely to report meeting the PA recommendations and have a higher prevalence of obesity than non-Latino whites [3,4] and these disparities may be due, at least in part, to differences in their neighborhood environments for supporting PA and health [5]. Yet, even among Latinos, there are subgroups who report disproportionately lower PA levels (e.g., leisure-time PA and total PA) including women, older adults, individuals with low education, and the less acculturated [6,7,8]. It is unknown whether these subgroups of less physically active Latinos report greater environmental inequities that are hindering PA. The current understanding of neighborhood environment perceptions among Latinos has been limited to studies focused on small geographic areas with limited diversity in environmental and population characteristics. A national study of Latino neighborhood environment perceptions by cultural and socio-demographic factors can help address this limitation and identify subgroups of Latinos reporting the greatest perceived environmental inequities hindering an active and healthy lifestyle.

Nationally, neighborhood environment perceptions are worse among certain socio-demographic groups [9,10]. For example, a study using nationally representative data from the 2015 National Health Interview Survey (NHIS) showed that older individuals and women reported fewer destinations in their neighborhoods [9]. That study also found that Latinos reported more built environment attributes (transportation infrastructure and destinations) in their neighborhoods than non-Latino whites [9]. However, grouping Latinos into a single category masks potential heterogeneity in neighborhood perceptions across socio-demographic (e.g., age, gender, and socio-economic status) and cultural subgroups such as by nativity status. Of these subgroups, nativity is the least understood in relation to neighborhood perceptions.

Nativity is a valid proxy measure of acculturation, which broadly reflects the process in which individuals change their attitudes, beliefs, and behaviors from their native culture to resemble that of their new cultural context [11]. Acculturation is related to health behaviors that are shaped by one’s environment, including diet and PA [12,13]. National studies show that among Latinos, higher acculturation (based on proxy measures such as nativity and language use) is related to higher leisure-time PA and lower transportation PA [8,14]. The exact reasons for such differences in PA by acculturation are not well understood but may be due in part to changes in socio-economic status, beliefs, and attitudes towards physical activity with increasing acculturation [8,14]. It is also possible that differences in neighborhood environment perceptions between cultural groups (e.g., U.S.-born vs. foreign-born) contribute to different PA domains. PA disparities among Latinos is of concern as it may contribute to health disparities. There is evidence that among Latinos in the U.S., U.S.-born Latinos often report higher prevalence of behavioral risk factors and worse health outcomes than foreign-born Latinos—a phenomenon known as the Latino health paradox [15,16]. However, it is unknown how environmental inequities between the U.S.- and foreign-born Latinos fit within this paradox. To date, only a handful of studies have examined how neighborhood environment perceptions vary across acculturation levels [17,18,19], and few have focused specifically on nativity or examined this question at the national level.

Of the handful of studies that have examined the relation between acculturation and neighborhood perceptions, findings have been mixed. One study with low-income, inner-city women (23% Latina) found that foreign-born women reported less perceived physical and social neighborhood disorder than U.S.-born women [20]. In two other studies that used a bi-dimensional acculturation scale, one study found that acculturation was not related to perceived safety and aesthetics [17] and the other study found that more acculturated Latinos reported fewer neighborhood environmental barriers than the less acculturated [18]. Although these studies are not directly comparable (e.g., differences in measures used and sample characteristics), they share some common limitations. Given that these studies were limited to specific geographic areas (single cities) and most included predominantly Mexican-American respondents, their findings are not generalizable to Latinos residing in other parts of the U.S. and those from other Latino backgrounds. As such, national studies are needed to address the limitations of past research. Further, additional studies with nativity status are needed as they may yield different associations with neighborhood environment perceptions than those observed with multidimensional scales.

Nativity may also interact with other individual characteristics to shape neighborhood environment perceptions. That is, neighborhood perceptions may be worse among those with multiple disadvantaged characteristics such as being an immigrant and of low socio-economic status (SES). Structural (e.g., SES) and contextual factors (e.g., experiences of specific gender or age groups) continually shape the acculturation experience [21]. However, we are not aware of any published studies examining interactions between nativity and socio-demographic factors in relation to neighborhood environment perceptions. Evidence of such interactions can help identify subgroups who perceive the greatest environmental inequities and could benefit from interventions.

Thus, the primary aim of the present study was to examine associations between nativity and neighborhood environment perceptions (outcomes)—including the presence of transportation infrastructure and destinations—among a nationally representative sample of Latinos. To examine how the interplay of the immigrant experience and socio-demographic factors contribute to neighborhood environment perceptions, the second aim was to test interactions of nativity with age, gender, and education.

## 2. Materials and Methods

### 2.1. Sample

Cross-sectional data came from a nationally representative sample of Latino adults (aged ≥ 18 years) from the 2015 National Health Interview Survey (NHIS). The NHIS is an annual in-person household survey that uses a multi-staged, probability sampling design to obtain a representative sample of the civilian, non-institutionalized U.S. population. Details of the survey design and interview methods are described in the Survey Description document [22]. A randomly selected adult from each household was asked to complete the NHIS core items (socio-demographics and health conditions) and the NHIS Cancer Control Supplement, which included a set of perceived neighborhood environment items. The National Center for Health Statistics Research Ethics Review Board approved all the NHIS activities and the respondents provided informed consent. Given that this study used publicly available deidentified data, it was exempt from Institutional Review Board review by the National Cancer Institute.

Of the 33,672 adults who completed the Cancer Control Supplement, 5591 self-identified as Latino or Hispanic (Central/South American, Cuban, Puerto Rican, Dominican Republic, or Spanish origin), hereafter labeled Latino. After excluding the 395 respondents who were missing at least one response on the perceived neighborhood environment items, the sample reduced to 5196 Latinos. The prevalence of missing environmental data was highest among foreign-born Latinos and those aged 25–34 years but did not differ by gender or education. Another 553 had missing data for the socio-demographic variables (*n* = 37), walking behaviors (*n* = 513), or both (*n* = 3), yielding a final analytic sample of 4643.

### 2.2. Measures

A team translation approach [23] involving professional translators, bilingual subject matter experts, and field interviewers translated the 2015 NHIS questionnaire items to Spanish followed by a similar approach used to translate the diet questions in the 2005 NHIS [24]. Approximately one-third of the analytic sample in the present study reported being mostly or only Spanish-speakers.

The Cancer Control Supplement included questions on the perceived presence (yes/no) of six environmental attributes falling under the broad categories of transportation infrastructure (2 items) and destinations (4 items) in the neighborhood (defined as “where you live”). The transportation infrastructure questions asked about “roads, sidewalks, paths, or trails” and “sidewalks on most streets”. The destination questions asked about “shops, stores, or markets;” “bus or transit stops;” “movies, libraries, or churches;” and “places that help you relax, clear your mind, and reduce stress” (shortened to places to relax). Interpretation of these perceived environment attributes was left to the respondents [9].

Because physically active individuals may be more aware of their environmental surroundings [25], we included self-report walking behaviors as covariates. The Cancer Control Supplement assessed walking for leisure (1 item) and transportation (1 item) in the past 7 days. Leisure walking was defined as walking for at least 10 min for any of the following reasons: for fun, relaxation, or to walk the dog, excluding walking for transportation. Transportation walking was defined as walking for at least 10 min to get some place.

The core NHIS items assessed the following socio-demographics: age (18–24, 25–34, 35–44, 45–64, and ≥65 years), gender (male or female), nativity status (foreign-born or U.S.-born), and education (‘less than high school’ or ‘high school/General Education Diploma (GED) or higher’). The age variable was further dichotomized based on the median age of 40 years (i.e., ‘less than 40′ or ‘40 and older’) for ease of interpretation in the interaction models. For any of the survey items, responses of “I don’t know” or declined to respond were treated as missing.

### 2.3. Analyses

We estimated the weighted proportions, and the corresponding 95% confidence interval (CI), for the socio-demographic, walking, and perceived neighborhood environment variables for the overall sample and by nativity. Chi-square tests assessed statistical differences between the nativity groups.

Multivariate logistic regression models examined the associations of nativity with each of the six perceived neighborhood environment attributes, controlling for the socio-demographic and walking variables (aim 1). To investigate the interplay between nativity and socio-demographic factors (aim 2), we tested two-way interaction terms between nativity and each socio-demographic variable (age, gender, and education) in relation to each perceived neighborhood environment attribute. We present the associations between the socio-demographic variables and each perceived neighborhood environment attribute stratified by nativity and controlling for the walking variables. We plotted interactions with *p*-values below 0.05. We did not make adjustments for multiple hypothesis testing.

Analyses were performed in SAS version 9.4 (SAS Institute, Cary, NC, USA) and SAS callable SUDAAN version 11.0.1 (Research Triangle Institute, Durham, NC, USA). All models used the sample weights and accounted for the complex survey design.

## 3. Results

### 3.1. Sample Descriptives

The analytic sample comprised 2733 foreign-born and 1910 U.S.-born Latinos (Table 1). In general, the foreign-born respondents were older in age and had less education than the U.S.-born respondents. There was about an equal balance of men and women. Approximately one-third of the sample reported any transportation walking and just under half reported any leisure walking in the past 7 days, with no significant differences observed by nativity.

Approximately 87% of the overall sample perceived roads, sidewalks, paths, or trails in their neighborhood, with a slightly higher proportion of U.S.-born respondents (89%) reporting such infrastructure than the foreign-born respondents (86%) (chi-square *p* = 0.004) (Table 1). Across the sample, the prevalence was also high for perceived sidewalks on most streets (80%) and all the destinations (each approximately 70%), except perceived movies, libraries, or churches, which was just over 50%.

### 3.2. Associations between Nativity and Neighborhood Environment Perceptions

Overall, nativity was not significantly associated with any of the six perceived neighborhood environment attributes in the adjusted models (Table 2). The unadjusted prevalence of perceived roads, sidewalks, paths, or trails was significantly higher among the U.S.-born than the foreign-born (prevalence ratio (PR) = 1.04, 95% CI: 1.01, 1.07), but this association became non-significant after controlling for the socio-demographic and walking variables (adjusted PR (APR) = 1.03, 95% CI: 1.00, 1.06).

### 3.3. Interactions between Nativity and Socio-Demographic Factors in Relation to Neighborhood Environment Perceptions

**Figure 1:**
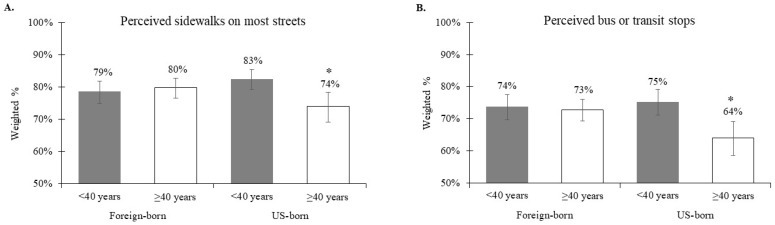
Interactions between nativity and age in relation to (**A**) perceived sidewalks on most streets (interaction *p* = 0.007) and (**B**) perceived bus or transit stops (interaction *p* = 0.01). Asterix indicates that the prevalence (95% CI) of the built environment attribute is significantly different by age for that nativity group.

Across the six neighborhood environment models, there were significant interactions between nativity and age in relation to perceived sidewalks on most streets (interaction *p* = 0.007; Figure 1A) and perceived bus or transit stops (interaction *p* = 0.01; Figure 1B). That is, older age was significantly related to lower prevalence of perceived sidewalks on most streets among the U.S.-born (APR = 0.91, 95% CI: 0.84, 0.98), while no association was found among the foreign-born (APR = 1.02, 95% CI: 0.97, 1.08) (Table 3; Figure 1A). Similarly, older age was significantly related to lower prevalence of perceived bus or transit stops among the U.S.-born (APR = 0.86, 95% CI: 0.79, 0.94), while no association was observed among the foreign-born (APR = 0.99, 95% CI: 0.93, 1.05) (Table 4; Figure 1B).

**Figure 2:**
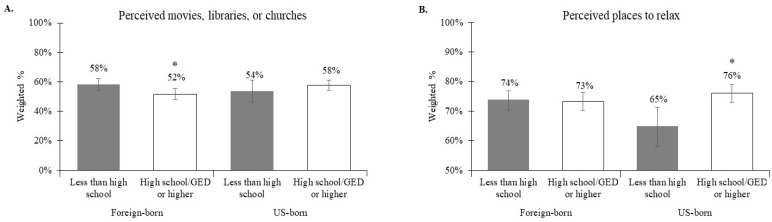
Interactions between nativity and education in relation to (**A**) perceived movies, libraries, or churches (interaction *p* = 0.03) and (**B**) perceived places to relax (interaction *p* = 0.008). Asterix indicates that the prevalence (95% CI) of the built environment attribute is significantly different by education for that nativity group.

There were also significant interactions between nativity and education in relation to perceived movies, libraries, or churches (interaction *p* = 0.03; Figure 2A) and perceived places to relax (interaction *p* = 0.008; Figure 2B). Higher education was significantly related to lower prevalence of perceived movies, libraries, or churches among the foreign-born (APR = 0.88, 95% CI: 0.80, 0.98), while no association was found among the U.S.-born (APR = 1.07, 95% CI: 0.92, 1.25) (Table 4; Figure 2A). In contrast, higher education was significantly related to higher prevalence of perceived places to relax among the U.S.-born (APR = 1.15, 95% CI: 1.03, 1.29), while no association was found among the foreign-born (APR = 0.99, 95% CI: 0.93, 1.05) (Table 4; Figure 2B).

## 4. Discussion

### 4.1. Summary of Findings

This is one of the first national studies to examine how neighborhood environment perceptions vary among Latinos across nativity and socio-demographic characteristics. This study addresses important limitations of past research that has focused on small geographic areas (e.g., west and southwestern cities in the U.S.), only one segment of the Latino population (Mexican-Americans), and have neglected the interplay of nativity with structural (e.g., SES) and contextual (e.g., gender and age) factors in shaping neighborhood perceptions. Overall, although our findings showed that nativity was not associated with neighborhood environment perceptions, interactions between nativity and age or education contributed to significant differences in perceptions of several built environment attributes (sidewalks; bus or transit stops; movies, libraries, or churches; and places to relax). Specifically, the greatest inequities (lowest perceptions) were found among disadvantaged U.S.-born Latinos (older or low education) and advantaged foreign-born Latinos (high education). These findings suggest that perceived neighborhood environment differences among Latinos are shaped by complex interactions between nativity and socio-demographic factors.

### 4.2. Main Effects of Nativity with Neighborhood Environment Perceptions

The main effects models showed that nativity was not associated with any of the perceived neighborhood environment attributes, after adjusting for the socio-demographic factors and walking behaviors. Such findings challenge previous studies with smaller samples that suggest neighborhood environment perceptions differ between foreign- and U.S.-born residents [5,20,26].

Findings from previous work that has examined the relationship between acculturation measures and neighborhood environment perceptions among Latinos have been mixed [17,18,27]. However, those studies were largely limited to sample populations residing in southwestern or west coast cities, which differ in the dominant Latino population represented (e.g., Mexican origin) and geographic landscape compared to other regions of the U.S. [9,28,29]. Our study used perceived neighborhood environment data from a nationally representative sample of Latinos, thereby enhancing variability in population and environmental characteristics.

The lack of differences in neighborhood perceptions between the U.S.- and foreign-born in our study could be explained by their possible neighborhood co-residence. That is, foreign- and U.S.-born Latinos (including children of immigrants) can reside in the same neighborhood and, thus, share a similar understanding of, and interaction with, their environments. Predominantly-Latino neighborhoods are growing in the U.S., especially in large cities, and those neighborhoods are comprised of an approximately equal balance of foreign- and U.S.-born Latinos [30].

### 4.3. Interactions between Nativity and Age in Relation to Perceived Sidewalks and Perceived Bus or Transit Stops

Although nativity was not a significant correlate of the perceived neighborhood environment attributes, our findings from the interaction models showed interactions between nativity and age. Specifically, older U.S.-born Latinos reported 9% lower prevalence of sidewalks and 14% lower prevalence of bus or transit stops in their neighborhoods compared to the younger U.S.-born. A possible explanation for this finding is that different age groups reside in neighborhoods that differ in urbanicity. That is, younger U.S.-born Latinos may reside in urban areas that are denser in population and have more sidewalks and public transportation. In contrast, older U.S.-born Latinos may reside in suburban or rural areas that are less dense and have fewer of these environmental attributes. A publication using data from the 2015 NHIS showed that compared to rural areas, urban areas were comprised of younger residents and had higher prevalence of perceived sidewalks and bus or transit stops. Although Latinos and immigrants are generally more concentrated in urban areas than suburban and rural areas [31], our findings suggest that older U.S.-born Latinos may be an exception and their less walkable neighborhoods may be placing them at increased risk of physical inactivity (e.g., lower transportation walking [1]).

### 4.4. Interactions between Nativity and Education in Relation to Perceived Movies, Libraries, or Churches and Perceived Places to Relax

Our interaction models also showed nativity by education interactions in relation to perceived movies, libraries, or churches and perceived places to relax, but the interactions were not always in the expected direction. That is, we found an unexpected negative association between education and perceived movies, libraries, or churches among the foreign-born. However, consistent with our expectations, there was a positive association between education and perceived places to relax among the U.S.-born.

A study using data from the 2015 NHIS showed that adults with higher education perceived more places to relax than those with low education [9], consistent with our finding among the U.S.-born Latinos. However, that study found no education differences for perceptions of movies, libraries, or churches [9]. Our results suggest that among Latinos, the interaction between nativity and education contribute to differences in perceptions of these neighborhood destinations. Potential explanations for these findings include differences in the urbanicity of the neighborhoods of different nativity and education groups [1] and personal preferences for neighborhoods with such destinations (i.e., neighborhood selection bias) [32].

Data from the 2015 NHIS show a higher prevalence of perceived movies, libraries, or churches as well as places to relax in urban compared to rural areas [1]. Further, individuals with a college degree or higher are more likely to reside in urban than rural areas [1]. However, among Latinos, often the foreign-born, who generally have lower education, are more concentrated in urban areas than the U.S.-born [31]. In our study, the foreign-born Latinos with less than a high school education may have resided in more dense urban areas with a higher presence of movies, libraries, or churches than those with higher education. In contrast, the foreign-born with higher education may have resided in less dense neighborhoods with fewer such destinations, e.g., suburban areas. As foreign-born individuals become more acculturated to the U.S., they may gain socio-economic resources (e.g., higher education and income) that allow them to become homeowners in neighborhoods such as the suburbs [33,34]. To our knowledge, there are no published national studies examining how assimilating to the U.S. influences residential selection among Latinos.

For perceived places to relax, the U.S.-born with higher education may have selected to live in neighborhoods with closer proximity to places to relax such as parks and other green spaces. However, this interpretation is in contrast to another national study that reported residents of lower SES neighborhoods (who are more likely to be of lower education) had greater access to parks than those in higher SES areas [35]. It is possible that respondents in our study interpreted ‘places to relax’ as those that were safe and in good-quality condition in addition to their presence. That is, those with lower education may have had places such as parks in their neighborhood, but they may not have found them relaxing if they perceived such places to be unsafe or in poor condition. Given that the NHIS neighborhood items were focused on perceptions, respondents’ interpretation of ‘places to relax’ could have varied across the sample. To better understand this finding, future work could examine how perceptions of safety are related to perceived places to relax and test if this relationship varies across education subgroups.

### 4.5. Strengths and Limitations

One limitation of this study is the cross-sectional design, which prevents causal inference. Given that acculturation is a process, longitudinal data (e.g., pre- and post-immigration) are needed to better understand how changes in acculturation influence neighborhood environment perceptions over time and in different residential contexts (e.g., following residential relocation). Further, we could not explore other acculturation and immigrant variables such as country of origin as they were not available in the NHIS public use dataset. The NHIS dataset is limited to unidimensional measures of acculturation and, as such, additional studies with different acculturation measures (e.g., those obtained by multidimensional acculturation scales) are needed to build on our study findings. Nevertheless, nativity is commonly used in Latino health research and population studies and is highly correlated with multidimensional acculturation scales [36,37,38].

Further, we did not have objective neighborhood environment data to assess the relative contributes of objective and perceived environment attributes. However, this is the topic of a future paper. The NHIS public use dataset also did not contain information on other potentially important confounders such as urbanicity and neighborhood immigrant concentration.

In our NHIS sample, it is possible that recent immigrants evaluated their U.S. neighborhood environments in relation to that of their native country, which may lead to biased perceptions. For example, a U.S. neighborhood with few sidewalks may receive a favorable evaluation from an immigrant who came from a rural area in their native country that had no sidewalks but a less favorable evaluation from an immigrant who came from a city in their native country with a high presence of sidewalks. Nevertheless, perceived built environment measures have been validated with Latino samples and have shown significant correlations with geographic information systems (GIS)-based measures, including in a Mexican adult sample [39].

A key strength of this study is that the sample is nationally representative, which provides greater heterogeneity in population and neighborhood environment characteristics compared to smaller studies. Such heterogeneity may have enhanced statistical power to detect associations and interactions. Further, we assessed a wide range of built environment attributes, including transportation infrastructure and destinations, which have unique contributions to PA behaviors [1].

## 5. Conclusions

Overall, we found that disadvantaged U.S.-born Latinos (older or low education) and advantaged foreign-born Latinos (high education) reported worse neighborhood environmental attributes. Future work linking the NHIS data to objective environment data is needed to complement these findings. Evidence from both perceived and objective environment research can help identify potential environmental targets for intervention. Changes to the actual environment may lead to changes in perceptions among Latinos and in turn, behavior change. To date, no such study has tested this potential mechanism in a Latino population. Further, differences in neighborhood environment perceptions, as shown in our study, may contribute to PA and health disparities among the Latino population in the U.S., a phenomenon known as the Latino Health Paradox [15]. To date, evidence on the Latino Health Paradox has predominantly focused on cultural and socio-demographic differences between U.S.- and foreign-born Latinos. However, our findings support the need to further investigate neighborhood environment inequities among Latinos, and the contributions of such inequities to health disparities across different nativity and socio-demographic groups.

Further, to better understand our findings, longitudinal research is needed examining neighborhood perceptions, attitudes, and preferences towards environmental attributes, as well as mobility patterns (e.g., international and domestic) among Latinos throughout the acculturation process. Such research may lend insight on the causal effects of cultural and environmental changes on Latino PA and health. Qualitative research may also provide in-depth understanding of how Latinos interpret different neighborhood environment attributes and why their perceptions of such attributes vary across nativity, age, and education. Such work can help identify potential personal (e.g., values and attachments) and social (e.g., cultural and economic) factors as well as prior experiences (e.g., growing up in a rural vs. urban environment) that could influence environment perceptions. Other countries experiencing international migration may need to examine how immigrants perceive their neighborhood environments and how this contributes to health disparities.

## Figures and Tables

**Table 1:** Weighted characteristics of Latino sample by nativity and overall, the NHIS 2015.

Characteristic	Foreign-Born	U.S.-Born	All
	(*n* = 2733)	(*n* = 1910)	(*N* = 4643)
	% (95% CI)	% (95% CI)	% (95% CI)
Socio-demographic			
Age (years) ^a^			
18–24	8.3 (6.9, 10.0)	30.9 (28.2, 33.7)	18.0 (16.5, 19.6)
25–34	19.8 (17.9, 21.8)	26.7 (24.1, 29.3)	22.8 (21.2, 24.3)
35–44	25.4 (23.3, 27.6)	16.0 (14.2, 18.0)	21.4 (19.9, 22.9)
45–64	34.4 (32.1, 36.8)	20.7 (18.3, 23.3)	28.5 (26.9, 30.1)
≥65	12.1 (10.7, 13.7)	5.8 (4.7, 7.2)	9.4 (8.4, 10.5)
Gender			
Male	50.4 (48.0, 52.8)	50.8 (48.0, 53.6)	50.6 (48.7, 52.4)
Female	49.6 (47.2, 52.0)	49.2 (46.4, 52.0)	49.4 (47.6, 51.3)
Education level completed ^a^			
Less than high school	44.4 (41.9, 46.9)	15.3 (13.5, 17.2)	31.9 (30.1, 33.7)
High school/GED or higher	55.6 (53.1, 58.1)	84.7 (82.8, 86.5)	68.1 (66.3, 69.9)
Walking ^b^			
Transportation	33.1 (30.9, 35.5)	30.0 (27.0, 33.1)	31.8 (29.9, 33.7)
Leisure	44.3 (41.7, 46.9)	47.3 (44.5, 50.2)	45.6 (43.7, 47.5)
Perceived neighborhood environment ^b^			
Transportation infrastructure			
Roads, sidewalks, paths, or trails ^a^	85.6 (83.4, 87.5)	89.2 (87.1, 91.1)	87.2 (85.4, 88.7)
Sidewalks on most streets	79.3 (76.6, 81.8)	79.7 (77.1, 82.2)	79.5 (77.5, 81.4)
Destinations			
Shops, stores, or markets	73.5 (70.7, 76.0)	73.4 (70.6, 76.1)	73.4 (71.5, 75.3)
Bus or transit stops	73.2 (70.2, 76.0)	71.6 (68.0, 75.0)	72.5 (70.0, 74.9)
Movies, libraries, or churches	54.7 (52.0, 57.3)	57.0 (54.0, 60.0)	55.7 (53.6, 57.7)
Places to relax	73.5 (71.1, 75.7)	74.4 (71.5, 77.0)	73.9 (72.1, 75.5)

Notes: CI = confidence interval; GED = General Education Diploma; NHIS = National Health Interview Survey. ^a^ Differences between nativity groups are statistically significant (Chi-square statistic *p* < 0.05). ^b^ Prevalence of ‘yes’ responses.

**Table 2:** Unadjusted and adjusted ^a^ prevalence ratios for perceived neighborhood environment attributes among Latinos by nativity, the NHIS 2015.

Nativity ^b^	Roads, Sidewalks, Paths, or Trails		Sidewalks on Most Streets		Shops, Stores, or Markets		Bus or Transit Stops		Movies, Libraries, or Churches		Places to Relax	
	PR (95% CI)	APR (95% CI)	PR (95% CI)	APR (95% CI)	PR (95% CI)	APR (95% CI)	PR (95% CI)	APR (95% CI)	PR (95% CI)	APR (95% CI)	PR (95% CI)	APR (95% CI)
Foreign-born	Ref	Ref	Ref	Ref	Ref	Ref	Ref	Ref	Ref	Ref	Ref	Ref
U.S.-born	**1.04** **(1.01, 1.07)**	1.03 (1.00, 1.06)	1.01 (0.96, 1.05)	0.98 (0.94, 1.03)	1.00 (0.95, 1.05)	0.99 (0.94, 1.04)	0.98 (0.92, 1.04)	0.98 (0.92, 1.04)	1.04 (0.97, 1.12)	1.05 (0.97, 1.13)	1.01 (0.96, 1.07)	0.99 (0.94, 1.04)

Notes: APR = adjusted prevalence ratio; CI = confidence interval; NHIS = National Health Interview Survey; PR = prevalence ratio. Bolded values are statistically significant. ^a^ Prevalence ratio adjusted for age, gender, education, transportation walking, and leisure walking. ^b^ Unweighted foreign-born *n* = 2733 and U.S.-born *n* = 1910.

**Table 3:** Multivariate associations of socio-demographic characteristics and walking behaviors with perceived neighborhood transportation infrastructure among Latinos, stratified by nativity, the NHIS 2015.

Characteristic	Roads, Sidewalks, Paths, or Trails		Sidewalks on Most Streets	
	% (95% CI)	APR (95% CI)	% (95% CI)	APR (95% CI)
Foreign-born				
Age (years)				
<40	85.0 (82.0, 87.6)	Ref	78.6 (74.9, 81.9)	Ref
≥40	86.0 (83.3, 88.3)	1.01 (0.98, 1.05)	79.8 (76.5, 82.7)	1.02 (0.97, 1.08)
Gender				
Male	86.8 (83.9, 89.2)	Ref	78.6 (74.7, 82.0)	Ref
Female	84.4 (81.6, 86.8)	0.97 (0.93, 1.01)	80.0 (76.8, 82.8)	1.01 (0.96, 1.06)
Education				
Less than high school	83.2 (80.0, 86.1)	Ref	76.9 (72.8, 80.6)	Ref
High school/GED or higher	87.4 (84.8, 89.7)	**1.05 (1.01, 1.10)**	81.2 (78.0, 84.0)	1.06 (1.00, 1.12)
Transportation walking				
None	84.1 (81.5, 86.4)	Ref	75.7 (72.4, 78.7)	Ref
Any	88.5 (85.3, 91.1)	1.04 (1.00, 1.08)	86.6 (83.0, 89.5)	**1.13 (1.08, 1.19)**
Leisure walking				
None	82.6 (79.5, 85.4)	Ref	76.1 (72.7, 79.2)	Ref
Any	89.3 (86.6, 91.5)	**1.07 (1.03, 1.12)**	83.4 (79.7, 86.5)	**1.07 (1.01, 1.13)**
U.S.-born				
Age (years)				
<40	89.5 (87.0, 91.6)	Ref	82.5 (79.3, 85.4)	Ref
≥40	88.7 (85.5, 91.2)	0.99 (0.96, 1.03)	74.0 (69.1, 78.3)	**0.91 (0.84, 0.98)**
Gender				
Male	89.0 (85.8, 91.5)	Ref	80.5 (76.9, 83.7)	Ref
Female	89.5 (86.8, 91.7)	1.00 (0.97, 1.04)	78.9 (75.2, 82.3)	0.99 (0.93, 1.05)
Education				
Less than high school	81.3 (74.6, 86.5)	Ref	73.2 (65.9, 79.5)	Ref
High school/GED or higher	90.7 (88.4, 92.5)	**1.12 (1.03, 1.20)**	80.9 (78.0, 83.5)	**1.11 (1.01, 1.23)**
Transportation walking				
None	87.6 (84.9, 89.9)	Ref	76.4 (72.9, 79.6)	Ref
Any	93.0 (89.8, 95.3)	**1.06 (1.02, 1.10)**	87.5 (83.4, 90.7)	**1.14 (1.07, 1.22)**
Leisure walking				
None	87.6 (84.4, 90.2)	Ref	78.7 (74.8, 82.1)	Ref
Any	91.1 (88.4, 93.2)	1.02 (0.98, 1.07)	80.9 (77.3, 84.1)	1.00 (0.94, 1.06)

Notes: APR = adjusted prevalence ratio; CI = confidence interval; GED = General Education Diploma; NHIS = National Health Interview Survey. Bolded values are statistically significant.

**Table 4:** Multivariate associations of socio-demographic characteristics and walking behaviors with perceived neighborhood destinations among Latinos, stratified by nativity, the NHIS 2015.

Characteristic	Shops, Stores, or Markets		Bus or Transit Stops		Movies, Libraries, or Churches		Places to Relax	
	% (95% CI)	APR (95% CI)	% (95% CI)	APR (95% CI)	% (95% CI)	APR (95% CI)	% (95% CI)	APR (95% CI)
Foreign-born								
Age (years)								
<40	75.8 (72.2, 79.1)	Ref	73.8 (69.6, 77.6)	Ref	57.2 (53.3, 61.0)	Ref	74.9 (71.6, 78.0)	Ref
≥40	71.9 (68.4, 75.1)	0.95 (0.90, 1.00)	72.8 (69.3, 76.0)	0.99 (0.93, 1.05)	53.0 (49.8, 56.1)	**0.92** **(0.85, 1.00)**	72.5 (69.4, 75.4)	0.96 (0.91, 1.02)
Gender								
Male	74.5 (70.7, 77.9)	Ref	74.6 (70.3, 78.5)	Ref	56.5 (52.6, 60.3)	Ref	74.4 (70.7, 77.7)	Ref
Female	72.4 (69.2, 75.4)	0.96 (0.91, 1.01)	71.8 (68.4, 74.9)	0.95 (0.90, 1.01)	52.8 (49.4, 56.3)	0.92 (0.84, 1.01)	72.5 (69.4, 75.5)	0.96 (0.90, 1.02)
Education								
Less than high school	74.3 (70.7, 77.5)	Ref	74.8 (71.2, 78.1)	Ref	58.4 (54.4, 62.3)	Ref	73.8 (70.4, 76.9)	Ref
High school/GED or higher	72.8 (68.9, 76.4)	0.98 (0.92, 1.05)	71.9 (67.7, 75.8)	0.96 (0.90, 1.03)	51.7 (48.0, 55.3)	**0.88** **(0.80, 0.98)**	73.2 (70.1, 76.2)	0.99 (0.93, 1.05)
Transportation walking								
None	67.9 (64.4, 71.2)	Ref	68.8 (65.2, 72.1)	Ref	49.3 (46.1, 52.5)	Ref	69.7 (66.7, 72.5)	Ref
Any	84.6 (81.2, 87.5)	**1.22** **(1.16, 1.30)**	82.1 (78.0, 85.7)	**1.18** **(1.11, 1.25)**	65.5 (61.8, 69.0)	**1.30** **(1.20, 1.40)**	81.1 (77.6, 84.1)	**1.12** **(1.06, 1.19)**
Leisure walking								
None	68.9 (65.3, 72.2)	Ref	70.1 (66.4, 73.6)	Ref	50.8 (47.3, 54.4)	Ref	65.8 (62.6, 68.9)	Ref
Any	79.2 (75.4, 82.6)	**1.12** **(1.05, 1.19)**	77.1 (73.0, 80.7)	**1.07** **(1.01, 1.14)**	59.5 (55.5, 63.3)	**1.13** **(1.03, 1.24)**	83.1 (79.9, 85.9)	**1.24** **(1.17, 1.32)**
U.S.-born								
Age (years)								
<40	77.1 (73.5, 80.3)	Ref	75.3 (71.1, 79.1)	Ref	58.5 (54.7, 62.2)	Ref	76.2 (72.7, 79.3)	Ref
≥40	65.9( 61.1, 70.3)	**0.87** **(0.80, 0.95)**	64.0 (58.5, 69.1)	**0.86** **(0.79, 0.94)**	54.0 (49.2, 58.8)	0.93 (0.84, 1.04)	70.7 (65.7, 75.2)	0.94 (0.87, 1.01)
Gender								
Male	76.0 (72.3, 79.3)	Ref	72.7 (67.7, 77.2)	Ref	57.4 (53.1, 61.6)	Ref	74.9 (70.2, 79.1)	Ref
Female	70.8 (66.1, 74.6)	0.95 (0.89, 1.02)	70.5 (66.1, 74.6)	0.99 (0.92, 1.07)	56.7 (52.6, 60.6)	1.00 (0.90, 1.10)	73.8 (69.9, 77.4)	0.98 (0.91, 1.06)
Education								
Less than high school	74.3 (67.8, 80.0)	Ref	72.7 (66.3, 78.2)	Ref	53.8 (46.4, 61.0)	Ref	65.0 (58.1, 71.3)	Ref
High school/GED or higher	73.3 (70.0, 76.3)	1.00 (0.91, 1.09)	71.4 (67.4, 75.1)	0.99 (0.91, 1.08)	57.6 (54.2, 61.0)	1.07 (0.92, 1.25)	76.1 (73.0, 78.9)	**1.15** **(1.03, 1.29)**
Transportation walking								
None	68.4 (64.7, 71.9)	Ref	66.8 (62.7, 70.7)	Ref	54.0 (50.3, 57.5)	Ref	70.7 (67.2, 74.0)	Ref
Any	85.2 (81.1, 88.5)	**1.23** **(1.14, 1.32)**	82.9 (77.6, 87.2)	**1.23** **(1.14, 1.32)**	64.2 (58.8, 69.2)	**1.16** **(1.05, 1.29)**	83.0 (78.5, 86.7)	**1.13** **(1.05, 1.21)**
Leisure walking								
None	71.2 (67.1, 75.0)	Ref	70.3 (65.6, 74.5)	Ref	53.7 (49.4, 57.9)	Ref	66.1 (62.1, 69.8)	Ref
Any	75.9 (72.3, 79.2)	1.03 (0.96, 1.10)	73.2 (68.1, 77.7)	1.00 (0.92, 1.09)	60.8 (56.4, 64.9)	1.10 (0.98, 1.22)	83.7 (80.4, 86.4)	**1.23** **(1.16, 1.31)**

Notes: APR = adjusted prevalence ratio; CI = confidence interval; GED = General Education Diploma; NHIS = National Health Interview Survey. Bolded values are statistically significant.
